# Composition, Antimicrobial, Anti-Inflammatory, and Potential Neuroprotective Activities of Volatile Oils in Solid Wood Boards from Different Tree Ages of *Cryptomeria japonica*

**DOI:** 10.3390/ijms26062400

**Published:** 2025-03-07

**Authors:** Churan Li, Boxiao Wu, Weihua Wang, Xiaoqin Yang, Xiaojian Zhou, Yingjun Zhang, Xiaoping Rao, Cheng Yang, Ping Zhao

**Affiliations:** 1 Key Laboratory of State Forestry and Grassland Administration on Highly-Efficient Utilization of Forestry Biomass Resources in Southwest China, Southwest Forestry University, Kunming 650224, China; churanli@swfu.edu.cn (C.L.); wbx1437@swfu.edu.cn (B.W.); 34016@ztu.edu.cn (W.W.); yangxiaoqin@swfu.edu.cn (X.Y.); xiaojianzhou@swfu.edu.cn (X.Z.); 2 Key Laboratory of Phytochemistry and Natural Medicines, Kunming Institute of Botany, Chinese Academy of Sciences, Kunming 650201, China; 3 Academy of Advanced Carbon Conversion Technology, Huaqiao University, Xiamen 361021, China; raoxp@hqu.edu.cn; 4 Key Laboratory of Green Chemistry & Technology, College of Chemistry, Sichuan University, Chengdu 610064, China; yangchengyc@scu.edu.cn

**Keywords:** solid wood board, *Cryptomeria japonica*, volatile oil, antifungal, anti-inflammatory, neuroprotective

## Abstract

The wood of *Cryptomeria japonica* (Japanese cedar or sugi) is widely used in building and adornment. This study aims to identify the composition of the volatile oils (VOs) extracted from *C. japonica* solid wood boards by gas chromatography–mass spectroscopy, and to investigate their antimicrobial, anti-inflammatory, and potential neuroprotective activities. A total of 58 volatile organic compounds (VOCs) were identified in the VOs from *C. japonica* solid wood boards with tree ages of 40, 50, and 60 years (VO-40, 50, and 60), with sandaracopimarinol (13.74–17.59%), ferruginol (10.23–11.29%), abieta-7,13-diene (8.20–13.66%), (+)-*δ*-cadinene (7.15–9.27%), *cis*-cubenol (4.36–6.36%), and sandaracopimarinal (3.23–6.04%) as major compounds. The VOs exhibited antifungal potential, especially VO-50 against *Aspergillus fumigatus* (MIC = 0.312 mg/mL), and VO-60 against *Gloeophyllum trabeum* (MIC = 1.25 mg/mL). However, VOs exhibited weak antibacterial activity (MIC > 10 mg/mL). Additionally, VOs (10 μg/mL) exhibited anti-inflammatory and potential neuroprotective activities, with VO-60 demonstrating the strongest inhibition of NO (25.79% reduction), TNF-*α* (52.24% reduction), and IL-6 (27.10% reduction) production in lipopolysaccharide-induced RAW264.7 cells, while increasing cell viability by 70.36% and reducing lactate dehydrogenase release by 41.10% in corticosterone-induced PC12 cells. Correlation and docking analyses revealed that sandaracopimarinal, sandaracopimarinol, *β*-eudesmol, and ferruginol were the potential active compounds. The results demonstrated that the volatile components from *C. japonica* solid wood boards not only enhance the board’s decay resistance, but also exhibit therapeutic potential for inflammatory disorders and neuropathic diseases.

## 1. Introduction

Indoor environmental conditions have a significant influence on human wellbeing. As people spend more time indoors for work and daily life, the importance of indoor air quality on the health of residents and workers has garnered significant attention. Inhabitants living in poorly built environments for a long time are susceptible to a variety of symptoms, such as the irritation of eyes, nose, or throat; headache; allergy; asthma; and allergic rhinitis, as well as insomnia, anxiety, depression, and exhaustion [1]. Therefore, the improvement of indoor air quality is crucial for ensuring the physical and mental health of individuals in both residential and workplace settings.

In rooms constructed from natural wood timber, the health and comfort of occupants have improved. Wooden indoor environments offer individuals comfortable sensory experiences and physiological relaxation through visual and tactile stimulation, thus benefiting occupants [2]. Specifically, the volatile organic compounds (VOCs) emitted from wood produce a distinctive aroma that positively affects human health. The aroma is believed to improve the physical, mental, and emotional states of humans, and is often used in aromatherapy, a complementary therapy that leverages essential oils for healing purposes [3]. The aromas of wood have been suggested to positively affect the mind and body of humans by stimulating the limbic lobe and hypothalamus [4]. VOCs emitted from wood include a variety of compounds, such as terpenoids, alcohols, aldehydes, and ketones [1]. These VOCs in certain woods possess various bioactivities, including a direct antibacterial effect on airborne pathogens, the alleviation of allergic rhinitis, and the suppression of negative emotional states, such as fatigue and depression [5,6,7]. Given these benefits of VOCs, utilizing woods that possess the ability to improve mental health in the construction of medical facilities has considerable potential.

*Cryptomeria japonica* (Thunb. ex L. f.) D. Don (Cupressaceae), known as Japanese cedar or sugi, is a plantation forest tree species that is distributed primarily in Japan, Korea, India, China, and Nepal. Its wood has garnered significant attention as a popular building material due to its straight-grained texture, light weight, and corrosion resistance. On the other hand, *C. japonica* is a source of bioactive metabolites that give its wood color, aroma, and resistance against termites and wood rot fungi [8]. VOCs emitted from *C. japonica* wood impart a special fragrance. Studies have reported that the VOCs emitted from *C. japonica* are dominated by terpenoids, with *α*-pinene the main one in leaves, and *δ*-cadinene in branches and stems [9]. *C. japonica* VOCs demonstrate environmental and health-promoting functions. For example, several studies show that the VOCs from *C. japonica* exhibit obvious anti-termitic and larvicidal activities, and repellent efficacy against mosquitos [10,11,12]. In addition, wood used for furniture that has decayed due to fungi emits spores and noxious odors, polluting the indoor environment, and consequently leading to health problems, such as respiratory diseases [13]. *C. japonica* VOCs demonstrate inhibitory effects against wood-rot fungi, pathogenic fungi, and bacteria, benefiting indoor air quality and human health [14,15,16]. Furthermore, the VOCs have a sedative effect on the autonomic nervous system and electroencephalogram [17]. Research has also indicated that the VOCs released from *C. japonica* in rooms have been reported to have a positive effect on human psychology and physiology [18,19].

Due to these unique aromatic qualities and beneficial VOCs, *C. japonica* wood has been positioned as a green building material for structural and interior construction. Solid wood boards made from *C. japonica*, which retain their original texture and natural aroma, are commonly used in furniture, flooring, and indoor decor production. While previous studies have explored the chemical composition of *C. japonica* VOCs, the relationship between tree age and VOC bioactivity remains poorly understood. Tree age is a crucial factor influencing VOCs release from wood [20]. Given the species’ widespread use in built environments, this study was aimed to systematically evaluate the chemical composition and bioactivity of volatile components emitted from *C. japonica* solid wood boards and how tree age (40, 50, and 60 years) affects them, with particular emphasis on their impacts on indoor environmental quality and human health. The volatile oils (VOs) were extracted from *C. japonica* solid wood boards with different tree ages, followed by the detection of their chemical composition and analysis of their antimicrobial, anti-inflammatory, and potential neuroprotective effects. Furthermore, molecular docking was employed to explore the potential pharmacological correlation of bioactive compounds.

## 2. Results and Discussion

### 2.1. Chemical Compositions of VOs from Solid Wood Boards with Different Tree Ages

The VOs of *C. japonica* solid wood boards with tree ages of 40, 50, and 60 years were extracted by supercritical CO_2_ fluid extraction (SFE-CO_2_), with the yields of VO-40, 50, and 60 being 1.14 ± 0.06%, 1.17 ± 0.07%, and 1.29 ± 0.05%, respectively. These yields were consistent with the VO yield reported in the literature for *C. japonica* wood [16], suggesting that a reliable extraction method was employed. The yields of VOs showed an increasing trend with increased tree age, and VO-60 showed the highest yield (1.29 ± 0.05%). Although the relationship between tree age and VOC release was unclear, a previous study also showed the highest yield of VOs from *C. japonica* leaves at an older tree age [21].

The VOs from different solid wood boards were further analyzed by gas chromatography–mass spectrometry (GC-MS). In the results, a total of 58 VOCs were identified in VOs of *C. japonica* solid wood boards with different tree ages by comparing them with retention indices (RI) and the MS database (Table 1). Notably, terpenoids constituted the largest group, with 50 species, including oxygenated monoterpenes by 4 species (0.01–0.02%), sesquiterpenes by 17 species (9.99–12.78%), oxygenated sesquiterpenes by 18 species (22.36–23.89%), diterpenes by 5 species (10.70–19.74%), and oxygenated diterpenes by 6 species (30.79–35.72%). In addition, there were two alcohols (0.01–0.02%), two aromatic hydrocarbons (0.29–0.39%), two hydrocarbons (0.004–0.01%), and two ketones (0.01–0.02%). Among them, sandaracopimarinol (**55**, 13.74–17.59%), ferruginol (**56**, 10.23–11.29%), abieta-7,13-diene (**51**, 8.20–13.66%), (+)-*δ*-cadinene (**23**, 7.15–9.27%), *cis*-cubenol (**36**, 4.36–6.36%), and sandaracopimarinal (**54**, 3.23–6.04%) were the predominant chemical compositions (average relative content > 5%). Following this, eight VOCs had an average relative content of 1–5%, and the top three VOCs were cubebol (**31**, 4.28–5.03%), (1*R*,4*R*,5*R*,6*R*,7*S*,10*R*)-4,10-dimethyl-7-propan-2-yltricyclo[4.4.0.01,5] decan-4-ol (**30**, 3.72–4.93%), and epicubenol (**38**, 3.27–4.87%). Furthermore, 44 VOCs were the trace components of VOs, with an average relative content of less than 1%, and the top three VOCs were cryptomerione (**46**, 0–1.00%), gleenol (**34**, 0.76–1.06%), and palustradiene (**49**, 0–0.69%).

Terpenoids, especially sesquiterpenes, were previously reported as the most abundant volatile components in *C. japonica* [9]. However, the results of the present study differ from these previous findings, indicating that diterpenes are also main compounds. Moreover, various studies have reported differing main compounds in the VOs extracted from *C. japonica* heartwood. Specifically, *δ*-cadinene, isoledene, cubebol, and epi-cubebol were identified as the main compounds [16,22]. These differences may be related to the different extraction methods used. Additionally, compounds **55**, **56**, **51**, and **23**, as the main volatile compounds of *C. japonica* solid wood board Vos, have garnered attention for their diverse bioactivities. Compound **55** was found to have antimicrobial and termiticidal activities [23,24]. Compound **56** also shows antimicrobial, antioxidant, ulcer healing, and termiticidal activities [24,25,26,27]. Compound **51** demonstrated antimalarial activity as an agent [28]. Compound **23** has been reported to exert analgesic, blood pressure-lowering, anti-inflammatory, antioxidant, antiallergic, antibacterial, and anticancer activities [29,30]. The bioactivities of these compounds emphasize the potential of *C. japonica* solid wood board VOs as a source of bioactive compounds with promising applications in therapy. Meanwhile, *C. japonica* solid wood board are suitable as the health-conscious building materials due to the beneficial volatile components they naturally contain.

**Figure 1:**
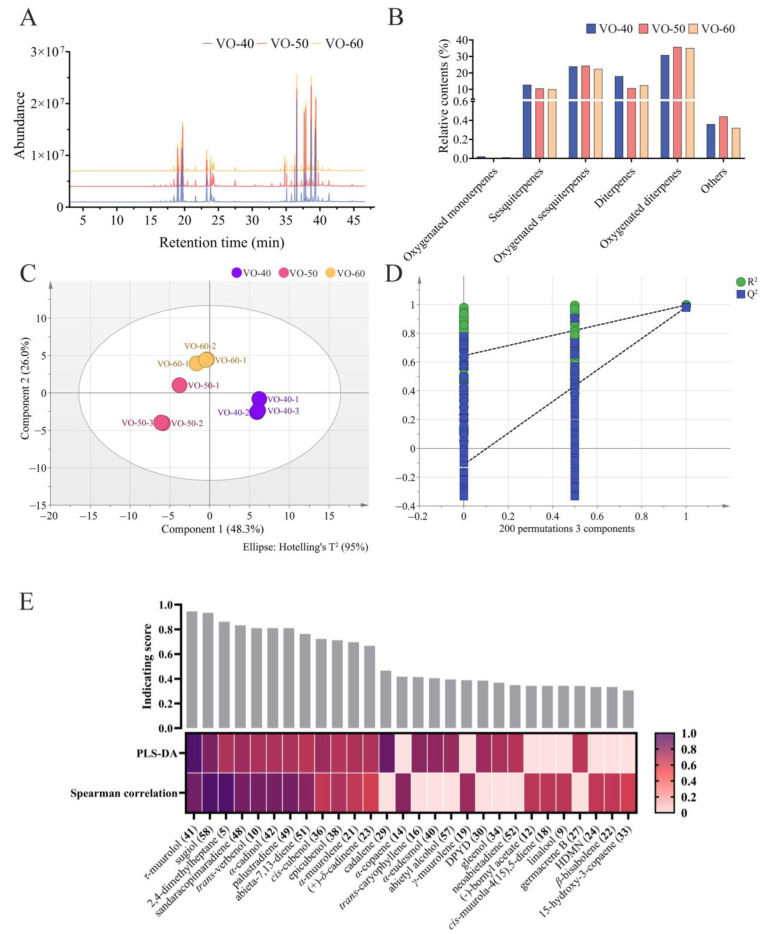
Chemical composition of VOs in *C. japonica* solid wood boards with different tree ages. (**A**) Total ions chromatograms of VO-40, 50, and 60. (**B**) Relative contents of each category of VOs. (**C**) PLS-DA score plot with the model parameter values of R^2^X (cum) = 0.825, R^2^Y (cum) = 0.994, Q^2^ (cum) = 0.927. (**D**) Permutation test plot with 200 iterations with intercepts of R^2^ = 0.644, Q^2^ = −0.113. (**E**) Heatmap of 28 potential differential VOCs selected by PLS-DA and Spearman’s correlation analysis, displaying VIP value and coefficient normalized by a min–max scaler. The histogram above the heatmap shows the indication scores of each VOC, calculated as the average of normalized VIP value and coefficient. Abbreviation: DPYD, (1*R*,4*R*,5*R*,6*R*,7*S*,10*R*)-4,10-di-methyl-7-propan-2-yltricyclo[4.4.0.01,5]decan-4-ol; HDMN, 1,2,3,4,4a,7-hexahydro-1,6-di-methyl-4-(1-methylethyl)-naphthalene.

The volatile components in VOs from *C. japonica* solid wood boards varied with different ages. The intensities and densities of peaks in total ions chromatograms for VO-40, 50, and 60 (Figure 1A). However, there was no significant relationship between tree age and content of VOCs (Figure 1B). Oxygenated diterpenes were the most abundant components in VOs, with higher relative contents of 35.72% and 35.11% in VO-50 and 60, respectively, and a lower relative content of 30.79% in VO-40 (Table 1 and Figure 1B). Oxygenated sesquiterpenes showed a high relative content in VO-50 (24.33%), followed by VO-40 (23.89%) and 60 (22.36%). In contrast, sesquiterpenes and diterpenes have the highest relative content in VO-40, with relative contents of 12.78% and 17.94%, respectively, and low relative contents in VO-50 and 60, with relative contents of 9.99–10.46% for sesquiterpenes and 10.70–12.40% for diterpenes. Additionally, oxygenated monoterpenes, alcohols, aromatic hydrocarbons, hydrocarbons, and ketones were the trace components, and their contents are not distinctly different in VO-40, 50, and 60.

**Figure 2:**
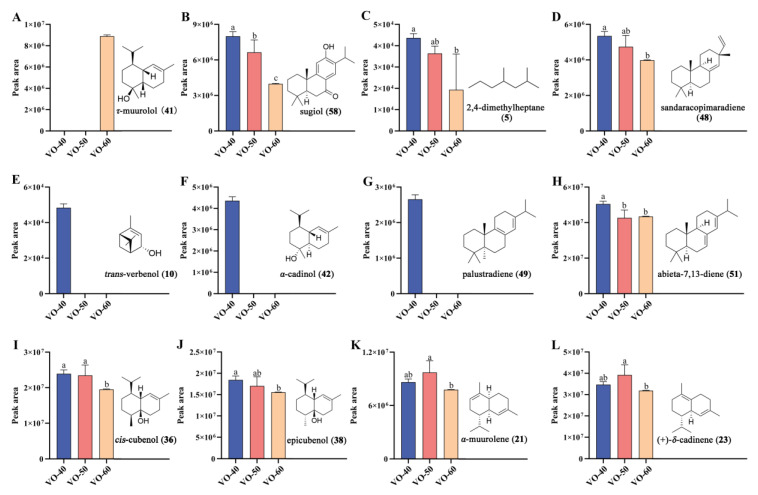
Peak area changes and chemical structures of 12 differential VOCs between VO-40, 50, and 60 in *C. japonica* solid wood boards. (**A**) *τ*-Muurolol. (**B**) Sugiol. (**C**) 2,4-Dimethylheptane. (**D**) Sandaracopimaradiene. (**E**) *Trans*-verbenol. (**F**) *α*-Cadinol. (**G**) Palustradiene. (**H**) Abieta-7,13-diene. (**I**) *Cis*-cubenol, (**J**) Epicubenol. (**K**) *α*-Muurolene. (**L**) (+)-*δ*-Cadinene. Different letters indicate significant differences at the 0.05 level.

To further identify the differential VOCs between VO-40, 50, and 60, partial least squares discriminant analysis (PLS-DA) and Spearman correlation analysis were performed [31]. The VO-40, 50, and 60 groups were obviously separated into three clusters based on their metabolic profiles in PLS-DA model (Figure 1C). A permutation test with 200 iterations was performed to evaluate the possible overfitting of the PLS-DA model. The results showed that the simulated values of R^2^ and Q^2^ are very close to one, indicating this model was effective and reliable (Figure 1D). Subsequently, the variable importance in projection (VIP) values of each VOCs were calculated based on the PLS-DA model. Twenty compounds with the highest VIP values were selected as candidate differential compounds (Table 2), with *τ*-muurolol (**41**), cadalene (**29**), and sugiol (**58**) as the top three compounds. The correlations between tree age and VOC expression were examined by Spearman correlation analysis. Similarly, twenty compounds with the highest Spearman correlation coefficients were selected as candidate differential compounds (Table 2), with **58**, 2,4-dimethylheptane (**5**), and **41** as the top three compounds. The above two multivariant analyses selected a total of 28 volatile compounds as potential differential compounds (Figure 1E). As a result, 12 VOCs were selected as differential VOCs, according to their indicating scores more than 0.5 and being selected by both methods. Among these, **58**, **5**, sandaracopimaradiene (**48**), **51**, **36**, and **38** showed reduced peak areas with increasing tree age. *α*-Muurolene (**21**) and **23** peaked in VO-50 (Figure 2). Additionally, *trans*-verbenol (**10**), *α*-cadinol (**42**), and palustradiene (**49**) were unique to VO-40, while **41** was only present in VO-60 (Figure 2).

### 2.2. Antifungal and Antibacterial Activity of VOCs in VOs from Solid Wood Boards

The VOs were applied to explore the antimicrobial activity against two mold fungi (*Aspergillus fumigatus* and *A. niger*), two wood-rotting fungi (*Gloeophyllum trabeum* and *Pyropolyporus fomentarius*), and three bacteria (*Staphylococcus aureus*, *Escherichia coli*, and *Pseudomonas aeruginosa*), by monitoring the mycelial growth status of fungi and bacteria and determining the minimum inhibitory concentration (MIC) (Table 3). The VOs demonstrated antifungal activity against four test fungi at higher concentrations (5.00 mg/mL). Specifically, the VO-50 exhibited superior antifungal activity for *A. fumigatus* (MIC = 0.312 mg/mL) and *A. niger* (MIC = 2.50 mg/mL) compared to other samples. Furthermore, the VO-60 were effective in inactivating *G. trabeum* (MIC = 1.25 mg/mL), while the VO-40 and 50 were effective in inactivating *P. fomentarius* (MIC = 2.50 mg/mL). However, the antifungal activity of ketoconazole (the positive control) was significantly stronger than that of the VOs. In addition, the VOs did not show antibacterial activity against the three tested bacteria (MIC > 10.00 mg/mL). Previous studies have indicated that *C. japonica* VOs exhibit weak antimicrobial activity against Gram-positive and Gram-negative bacteria, in contrast to the potent inhibition of mycelial growth of wood-rotting fungi [9,15,32].

Terpenoids are considered to possess antibacterial activity, and are capable of inhibiting fungal growth and germination. Previous research suggests that terpenoids may disrupt the integrity of fungal cell walls and block fungal metabolic pathways by inhibiting the activity of enzymes within fungal cells, including acid phosphatase, chitinase, and protease, thereby exerting an antifungal effect [33]. Terpenoids present in *C. japonica* solid wood boards VOs, such as *β*-eudesmol (**39**) and ferruginol (**56**), may be responsible for their antifungal activity [34]. The observed weak antibacterial activity of *C. japonica* solid wood boards VOs may be attributed to the hydrophilic channels in the cell walls of Gram-negative bacteria, which exclude the passage of lipophilic compounds, while the thick peptidoglycan layer in Gram-positive bacteria provides greater resistance [35]. The current study demonstrates that the VOCs present in *C. japonica* solid wood boards retain their natural antifungal activity, thereby conferring potential antifungal properties to these processed wood products.

### 2.3. Anti-Inflammatory Activity of VOCs in VOs from Solid Wood Boards

**Figure 3:**
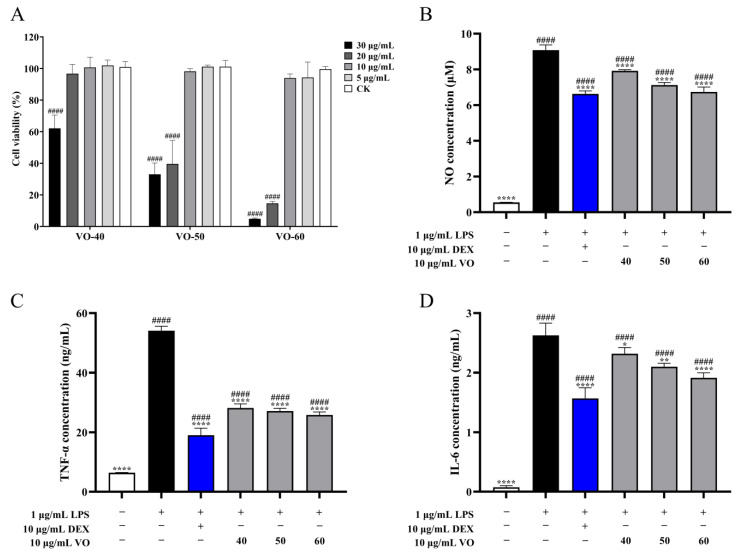
Anti-inflammatory activity of VOs in *C. japonica* solid wood boards with different tree ages. (**A**) Cytotoxicity of VOs in RAW264.7 cells. (**B**) Effects of VOs on NO production in LPS-induced RAW264.7 cells. (**C**) Effects of VOs on TNF-*α* production in LPS-induced RAW264.7 cells. (**D**) Effects of VOs on IL-6 production in LPS-induced RAW264.7 cells. Data are presented as the mean ± SD, *n* =3. * *p* < 0.05, ** *p* < 0.01, and **** *p* < 0.0001 versus LPS group; #### *p* < 0.0001 versus the control group.

The cytotoxicity of VOs on RAW264.7 cells showed that VO-40, 50, and 60 had no effect on cell viability at concentrations below 10 μg/mL (Figure 3A). Cell viability decreased significantly (*p* < 0.0001) in cells treated with VOs beyond 10 μg/mL (Figure 3A). Therefore, 10 μg/mL VOs were used in subsequent experiments. To evaluate the anti-inflammatory activity of VOs, the levels of nitric oxide (NO), tumor necrosis factor-*α* (TNF-*α*), and interleukin 6 (IL-6) in lipopolysaccharide (LPS)-induced RAW264.7 cells was determined. Treatment with1 μg/mL LPS significantly (*p* < 0.0001) increased the NO concentration to 9.07 ± 0.30 μM from 0.55 ± 0.01 μM (control group) (Figure 3B). Meanwhile, treatment with 10 μg/mL VOs inhibited the production of NO. NO levels of 7.93 ± 0.07, 7.12 ± 0.15, and 6.73 ± 0.27 μM of VO 40-, 50-, and 60-treated cells, reducing by 12.64%, 21.53%, and 25.79% compared to the LPS group, respectively (Figure 3B). Furthermore, LPS-induced RAW264.7 cells markedly up-regulated (*p* < 0.0001) the production of TNF-*α* and IL-6, with their concentrations of 54.07 ± 1.52 and 2.63 ± 0.21 ng/mL, respectively. The VO-40, 50, and 60 inhibited 47.96%, 49.78%, and 52.24% of TNF-*α* production, respectively, as well as 11.75%, 20.10%, and 27.10% of IL-6 production (Figure 3C,D).

Exposure to the harmful and polluted indoor environment provokes irritation of the respiratory system, causing allergic rhinitis, asthma, and other prevalent inflammatory diseases of the respiratory airways [6]. LPS is a common inducer that can stimulate macrophages and interact toll-like receptors to establish the inflammatory model [36]. The nuclear factor kappa-B and phosphorylation of mitogen-activated protein kinases were activated in stimulated macrophages, leading to the production of inflammatory mediators (NO, prostaglandin E2) and cytokines (IL-6, TNF-*α*, and IL-1*β*) [37]. NO plays an important role in the modulation of inflammation [38]. TNF-*α* and IL-6 are important pro-inflammatory cytokines that have been associated with the pathogenesis of many infectious and inflammatory diseases [39]. It is considered that drugs-inhibiting mediators and pro-inflammatory cytokines production can be used for the treatment of inflammation. This study demonstrates that VOs from *C. japonica* solid wood boards, particularly VO-60, has anti-inflammatory activity by inhibiting the LPS-induced secretion of mediators (NO) and pro-inflammatory cytokines (TNF-*α* and IL-6) in RAW264.7 cells. The anti-inflammatory property of volatile components from *C. japonica* solid wood boards suggests their potential in the treatment of allergic rhinitis and asthma, and highlights their status as green building materials which are beneficial to human health.

### 2.4. Potential Neuroprotective Activity of VOCs in VOs from Solid Wood Boards

**Figure 4:**
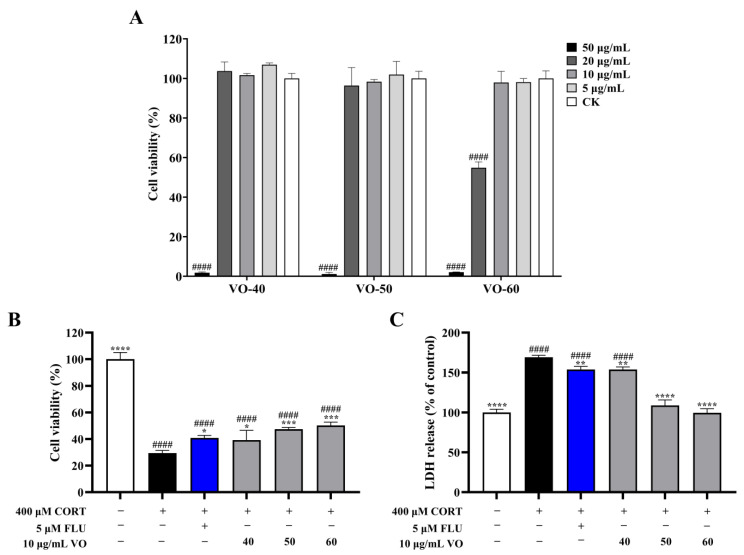
Potential neuroprotective activity of VOs in *C. japonica* solid wood boards with different tree ages. (**A**) Cytotoxicity of VOs on PC12 cells. (**B**) Effects of VOs on cell viability in CORT-induced PC12 cells. (**C**) Effects of VOs on LDH release in CORT-induced PC12 cells. Data are presented as the mean ± SD, *n* = 3. * *p* < 0.05, ** *p* < 0.01, *** *p* < 0.001, and **** *p* < 0.0001 versus CORT group; #### *p* < 0.0001 versus the control group.

The cytotoxicity of VOs on PC12 cells revealed that VO-40, 50, and 60 had no effect on cell viability at concentrations below 10 μg/mL (Figure 4A), which were used to treat the corticosterone (CORT)-induced neurotoxicity in PC12 cells. Treatment with 400 μM CORT significantly decreased (*p* < 0.0001) cell viability to 29.50 ± 1.97%, but the VOs exerted protective effects against CORT-induced cytotoxicity (Figure 4B). The treatment of VO-40, 50, and 60 increased cell viability to 39.21 ± 7.33%, 47.41 ± 1.39%, and 50.25 ± 2.50%, respectively. Furthermore, CORT (400 μM) treatment resulted in increased lactate dehydrogenase (LDH) release in PC12 cells by 69.03% compared to the control group (Figure 4C). Treatment with VO-40, 50, and 60 inhibited LDH release in cells with LDH releases by 8.99%, 35.52%, and 41.10% compared to CORT group (Figure 4C).

Differentiated PC12 cells are neuron-like cells with axons similar in structures and functions to neuronal cells, which are widely used in neuropathic diseases such as depression. As an end product of the hypothalamic–pituitary–adrenal axis, CORT can induce the neurotoxicity of PC12 cells. Increasing evidence suggests that neurogenesis plays a crucial role in the development of depression and the therapeutic effects of antidepressants [40]. The volatile components in plants have been found to mitigate stress symptoms and treat symptoms of mental health [41]. Previous research has shown that VOCs emitted from *C. japonica* woods had a positive effect on the psychophysiology of humans [19]. These results suggested the VOs from *C. japonica* solid wood boards have potential neuroprotective activity, supporting previous studies. It is also indicated that the use of *C. japonica* solid wood boards in construction may help relieve stress and promote mental health.

### 2.5. Correlation Analysis of Chemical Component in VOs with Their Activities

**Figure 5:**
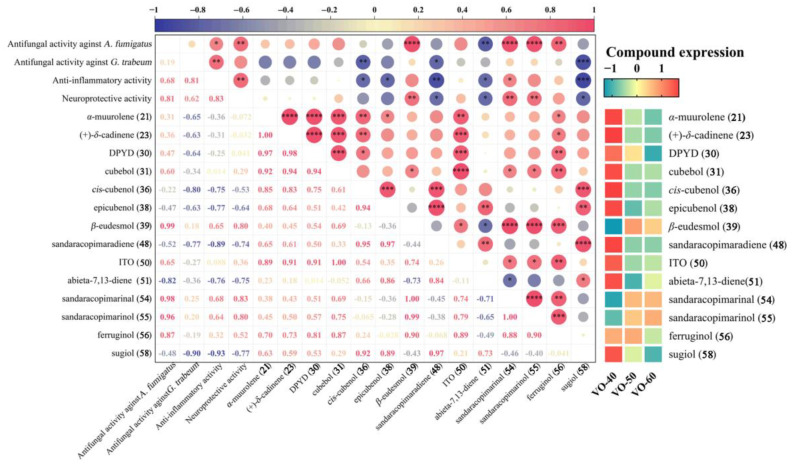
Correlation analysis of antifungal activity with compounds with relative content > 1% of VOs, with compounds expression. Asterisk symbols (*) indicate significance (* *p* < 0.05, ** *p* < 0.01, *** *p* < 0.001, and **** *p* < 0.0001). Abbreviation: DPYD, (1*R*, 4*R*,5*R*,6*R*,7*S*,10*R*)-4,10-dimethyl-7-propan-2-yltricyclo[4.4.0.01,5]decan-4-ol; ITO, 7-isopropyl-1,1,4a-trimethyl-1,2,3,4,4a,9,10,10a-octahydrophenanthrene.

Pearson’s correlation analysis was carried out to investigate the potential relationship between the compounds with relative content > 1% of VOs with their antifungal, anti-inflammatory, and potential neuroprotective activities (Figure 5). The VOs showed good antifungal activity against *A. fumigatu* and *G. trabeum*, and the correlation analysis was used to identify the potential active compounds. The antifungal activity of VOs against *A. fumigatus* showed a significantly positive correlation with *β*-eudesmol (**39**), sandaracopimarinal (**54**), and sandaracopimarinol (**55**) (*p* < 0.0001), and a strong correlation with ferruginol (**56**) (*p* < 0.01). The antifungal activity of VOs against *G. trabeum* was positively correlated with **54**, **55**, and **39** (*p* > 0.05), but their correlation coefficients were low. This suggests that the primary influence is from synergistic effects of multiple compounds, rather than a single main compounds. Furthermore, the anti-inflammatory and potential neuroprotective activities of VOs were also positively correlated with **54**, **55**, **39**, and **56**, especially with significant correlations between the anti-inflammatory activity and **54** (*p* < 0.05), and significant correlations between the potential neuroprotective effect and **54**, **55**, and **39** (*p* < 0.01).

Overall, compounds **54**, **55**, **39**, and **56** showed strong positive correlations with antifungal activity against *A. fumigatus* and *G. trabeum*, as well as anti-inflammatory and potential neuroprotective properties (Figure 5). These compounds have higher expression in VO-50 and 60 (Figure 5), which possessed strong activities. Compounds **54**, **55**, **39**, and **56** were the chemical components reported in *C. japonica* for their antimicrobial activity [8]. Previously, research has found that compound **56** exhibits antioxidant and beta-amyloid (A*β*) toxicity reduction effects [42], while compound **55** inhibits A*β* aggregation and protects PC12 cells [43], potentially making them useful for treating Alzheimer’s disease. In addition, compound **56** has been reported to have miticidal, cardioactive, anti-oxidative, anti-plasmodial, anti-inflammatory, and anti-ulcerogenic properties [30,44]. Moreover, compound **39** also exhibits anticancer, improving nervous system, anti-inflammatory, and anti-allergic activities [45]. Thus, these compounds in VOs from *C. japonica* solid wood boards may partially account for their antifungal, anti-inflammatory, and potential neuroprotective activities.

### 2.6. Molecular Docking Analysis

Molecular docking serves as a computational tool to elucidate the intricate molecular mechanisms of compounds with pharmacological activity [46]. In this study, molecular docking was used to further explore the potential mechanisms of antifungal, anti-inflammatory, and potential neuroprotective activities with sandaracopimarinal (**54**), sandaracopimarinol (**55**), *β*-eudesmol (**39**), and ferruginol (**56**) in VOs. The re-docking analysis of the co-crystallized inhibitors to the protein targets confirms that the RMSD values for each pose of the co-crystallized inhibitors are below 2.00 Å, indicating the reliability and accuracy of the docking scheme. As shown in Table 4, a total of 25 pairs of ligand-protein interaction were investigated, in which binding energies less than −5.0 kcal/mol indicated that the ligand had a strong binding capacity to the protein.

**Figure 6:**
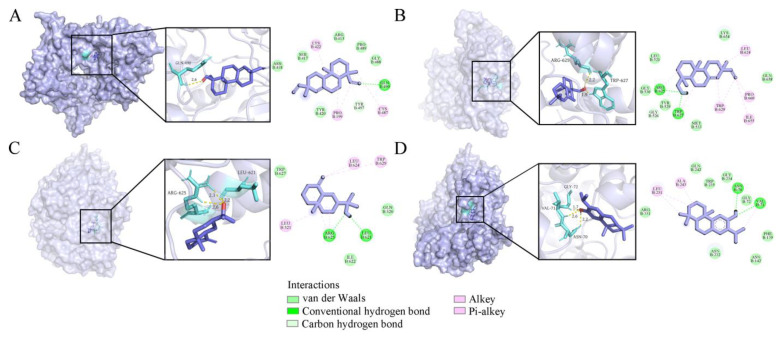
Four compounds docked with chitin synthase. (**A**) Sandaracopimarinol. (**B**) Sandaracopimarinol. (**C**) *β*-Eudesmol. (**D**) Ferruginol. From left to right, overall 3D, partial SD, and 2D images in order. Light purple, acro-molecules; purple, active compounds; aquamarine, amino acid residues around the binding bag; yellow dashed lines, hydrogen bonds in 3D image.

Chitin is an important component of the fungal cell wall and is an attractive target for fungicides due to it being absent in plants and mammals. Hence, chitin synthase is a crucial target for selective inhibitors against fungi [47]. The binding modes of compounds **54**, **55**, **39**, and **56** with chitin synthase (PDB ID: 4WJW) were modeled by molecular docking, and the binding energies of the four ligands to chitin synthase were calculated as −5.64, −6.72, −5.24, and −6.35 kcal/mol, respectively (Figure 6 and Table 4). Compound **55** was found to have the highest inhibitory potential against chitin synthase with the lowest binding energy of −6.72 kcal/mol, and was capable to form two conventional hydrogen bonds with ARG 625 and TRP 627 amino acid residues (Figure 6B).

**Figure 7:**
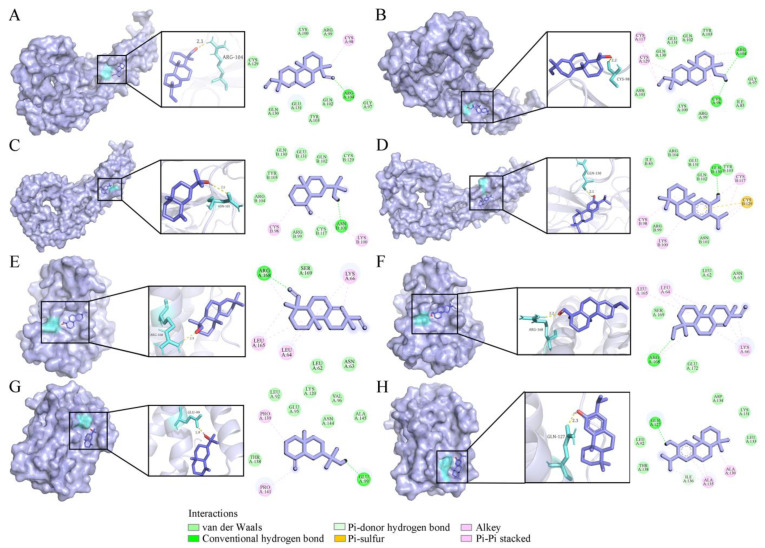
Four compounds docked with TNF-*α* and IL-6. (**A**) Sandaracopimarinol with TNF-*α*. (**B**) Sandaracopimarinol with TNF-*α*. (**C**) *β*-Eudesmol with TNF-*α*. (**D**) Ferruginol with TNF-*α*. (**E**) Sandaracopimarinol with IL-6. (**F**) Sandaracopimarinol with IL-6. (**G**) *β*-Eudesmol with IL-6. (**H**) Ferruginol with IL-6. From left to right, overall 3D, partial 3D, and 2D images in order. Light purple, acro-molecules; purple, active compounds; aquamarine, amino acid residues around the binding bag; yellow dashed lines, hydrogen bonds in 3D image.

VOs have been found to possess anti-inflammatory activity by inhibiting the productions of TNF-*α* and IL-6. As the important pro-inflammatory cytokines [37], TNF-*α* (PDB ID: 1EXT) and IL-6 (PDB ID: 1ALU) were used for molecular docking analysis (Figure 7). Four compounds showed binding energies ranging from −5.31 to −6.41 kcal/mol with TNF-*α* (Table 4). Compound **55** was the most favorable molecule, with a binding energy of −6.41 kcal/mol. It had two conventional hydrogen bonds with CYS 98 and ARG 101 amino acid residues (Figure 7B). For the IL-6, the binding energies of compounds **54**, **55**, **39**, and **56** were −6.69, −5.41, −5.43, and −5.96 kcal/mol, respectively (Table 4). Compound **54** was the strongest inhibitor against IL-6 exerting one conventional hydrogen bond with amino acid residue ARG 168 (Figure 7E).

**Figure 8:**
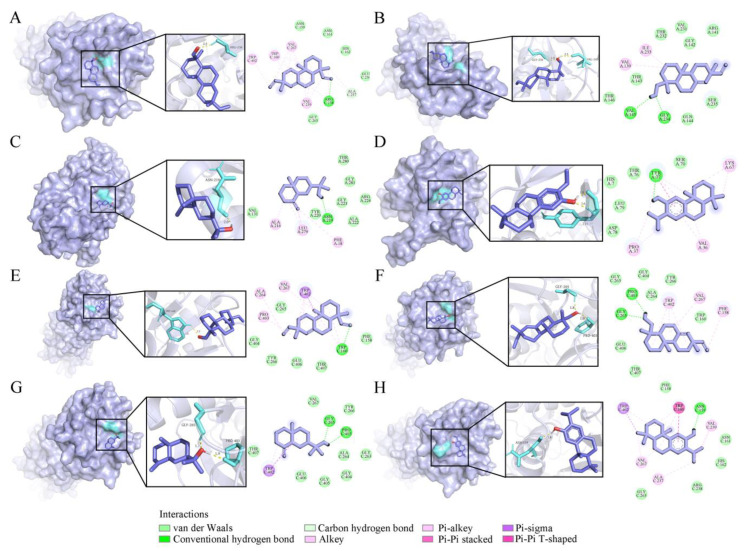
Four compounds docked with GluN 1 and GluN 2B. (**A**) Sandaracopimarinol with GluN 1. (**B**) Sandaracopimarinol with GluN 1. (**C**) *β*-Eudesmol with GluN 1. (**D**) Ferruginol with GluN 1. (**E**) Sandaracopimarinol with GluN 2B. (**F**) Sandaracopimarinol with GluN 2B. (**G**) *β*-Eudesmol with GluN 2B. (**H**) Ferruginol with GluN 2B. From left to right, overall 3D, partial 3D, and 2D images in order. Light purple, acro-molecules; purple, active compounds; aquamarine, amino acid residues around the binding bag; yellow dashed lines, hydrogen bonds in 3D image.

VOs also exhibited potential neuroprotective activity, suggesting their potential in the treatment of neurological diseases. The medicines for treating depression are alleviate symptoms through interacting with *N*-methyl-*D*-aspartate receptors, including GluN1 and GluN2B receptor subunits. In particular, GluN2B is related to many neurological disorders [48]. Therefore, in this study, GluN1 (PDB ID: 5H8Q) and GluN2B (PDB ID: 5EWM) were selected as the protein targets (Figure 8). Except for compound **55** (−4.99 kcal/mol), the binding energies of the other three compounds with GluN1 were lower than −5.0 kcal/mol. Compound **56** possessed the lowest binding energy (−6.14 kcal/mol), followed by **54** (−5.95 kcal/mol) and **39** (−5.58 kcal/mol) (Table 4). Compound **56** has stronger binding affinity, exerting one conventional hydrogen bond with amino acid residue TYR 77 (Figure 8D). For GluN2B, the binding energy of four compounds ranged from −5.00 to −6.04 kcal/mol, with compound **54** being the strongest inhibitor exerting one conventional hydrogen bond with amino acid residue TRP 160 (Figure 8E).

## 3. Materials and Methods

### 3.1. Samples and Preparation

*C. japonica* solid wood boards used in this study were sourced from Okayama, Japan and provided by the Japanese Wood Products Export Association. These boards were manufactured using heartwood obtained from trees aged 40, 50, and 60 years. All samples were ground into meal (40-mesh) and stored in sealed bags at 4 °C in the dark for subsequent analysis.

### 3.2. Extraction of VOs

VOs from the board samples with tree ages of 40, 50, and 60 years (VO-40, 50, and 60) were extracted using SFE-CO_2_. The powders (1.0 kg each) of solid wood boards from three different tree ages were extracted and performed on a Speed SFE-2 apparatus (Applied Separations, Allentown, PA, USA) with CO_2_ in a supercritical extraction vessel at a pressure of 30 MPa and a temperature of 40 °C. The VOs were obtained after static extraction for 30 min and then dynamic extraction with CO_2_ flow rate of 10 L/min for 1 h. The yields of VOs from three samples were 11.35, 11.66, and 12.88 g, respectively. The obtained VOs were stored at 4 °C for further analysis.

### 3.3. GC-MS Analysis of VOs

The VO (10 μL) was dissolved in 990 μL of *n*-hexane (Merck, Darmstadt, Germany), filtered, and then injected into an injector of GC-MS (7090B/5977B, Agilent, Santa Clara, CA, USA) equipped with an HP-5MS capillary column (10 m × 0.25 mm × 0.25 μm film thickness, Agilent, Santa Clara, CA, USA). Helium served as the carrier gas at a constant flow of 1 mL/min. The inlet temperature was 250 °C, with the following temperature program: initial temperature at 40 °C, increased to 90 °C at a rate of 15 °C/min, then increased at 5 °C/min to 150 °C and held for 5 min, followed by being increased to 160 °C at 2 °C/min and held for 3 min, and subsequently increased up to 240 °C at 15 °C/min and held for 10 min. The mass spectrometer was used in electron ionization (70 eV) in scan mode over a range of *m*/*z* 35–550 with the temperature of the ion source set as 230 °C.

The VOCs were identified by comparison of the mass spectra of database and RIs. The mass spectra of peaks were compared with the NIST 14 library, using a match score of <80% as a cut-off. The RIs of compounds were calculated by a series of *n*-alkanes (C_7_–C_40_) and compared to KIs reported in NIST Chemistry WebBook (http://www.nist.gov, accessed on 5 November 2024) and the published literature [49,50]. To determine the relative content of each sample component, the percentage peak area of each compound was calculated relative to the total peak area of the chromatogram. Total area normalization for each sample was performed, and further used for multiple variable analysis. PLS-DA was performed using the software SIMCA 14.1 (Umetrics, Umea, Sweden), and Spearman correlation analysis was implemented in MetaboAnalyst (http://www.metaboanalyst.ca, accessed on 5 November 2024).

### 3.4. Antimicrobial Activity of VOs from C. japonica Solid Wood Boards

#### 3.4.1. Fungal and Bacterial Strains

Antimicrobial activity of VOs from *C. japonica* solid wood boards was investigated against four fungi, namely, *A*. *niger* (CICC 40373), *A*. *fumigatus* (CICC 40537), *G*. *trabeum* (CFCC 86617), and *P*. *fomentarius* (CGMCC 5.1083), as well as three bacteria, namely, *S*. *aureus* (CICC 10788), *E*. *coli* (CICC 10899), *P*. *aeruginosa* (CICC 20236).

#### 3.4.2. MIC

The tested fungal strains were cultured in potato sucrose agar medium at 28 °C for 5–7 days, whereas tested bacterial strains were cultured in Luria–Bertani broth at 37 °C for 12 h, and shaken at 149 rpm. Following this, fungal spores on the plates were dissolved in sterile saline and bacterial cultures were diluted in Luria–Bertani medium. The turbidities of fungal and bacterial suspension were amended following McFarland standards 0.5 [51]. The MIC determination of VOs was performed using the two-fold serial dilution method. All antimicrobial analyses were conducted in potato sucrose broth for fungi and in Luria–Bertani broth for bacteria. Dimethyl sulfoxide (DMSO, 5%, *v*/*v*), used as an emulsifier was, respectively, dissolved in potato sucrose broth and Luria–Bertani broth, and then used to make a serial dilution of VOs (0.039, 0.078, 0.156, 0.312, 0.625, 1.25, 2.50, 5.00, 10.00, and 20.00 mg/mL). Subsequently, 75 μL of the broth containing specified concentrations of VOs was added to each well of a 96-well plate, and 75 μL of fungal or bacterial suspension was transferred to each well. The final concentrations of VO in reaction system were 0.0195, 0.039, 0.078, 0.156, 0.312, 0.625, 1.25, 2.50, 5.00, and 10.00 mg/mL, respectively. And the ultimate concentration of DMSO in the well was maintained at a level below 5% (preliminary analyses with 5% (*v*/*v*) DMSO/broth affected neither the growth of the test fungi nor bacteria). Ketoconazole and tetracycline served as positive controls. The negative control consisted of broth containing 5% (*v*/*v*) DMSO and fungal or bacterial suspension, and the blank control consisted of broth and fungal or bacterial suspension. The plates were covered with a sterile plate sealer, then incubated at 28 °C for antifungal tests and at 37 °C for 12 h for antibacterial tests. The MIC was the lowest concentration at which no fungi and bacteria growth was observed in any well of the 96-well plate [52].

### 3.5. Anti-Inflammatory Activity of VOs in LPS-Induced RAW264.7 Cells

RAW264.7 cells were cultured in Dulbecco’s modified Eagle’s medium (DMEM) (Pricella, Wuhan, China) supplemented with 10% fetal bovine serum (FBS) (Pricella, Wuhan, China) and 1% penicillin–streptomycin antibiotics at 37 °C in a 5% CO_2_-humidified incubator. RAW264.7 cells (1 × 10^5^ cells/mL) were cultured in 96-well plates for 24 h. The cells were treated with VOs at a concentration of 0–50 μg/mL for 24 h to evaluate their cytotoxicity with a Cell Counting Kit-8 (CCK-8) assay.

To determine the anti-inflammatory activity of VOs, RAW264.7 cells (1 × 10^5^ cells/mL) were seeded in 96-well plates and cultured for 24 h. Then, they were subcultured into four groups: control group: without any treatment, LPS group: treated with 1 μg/mL LPS, experiment group: treated with 10 μg/mL VOs (VO-40, 50, and 60) and 1 μg/mL LPS, positive control group: treated with 10 μg/mL dexamethasone (DEX) and 1 μg/mL LPS. After treatment for 24 h, the concentrations of NO, TNF-*α*, and IL-6 in culture supernatants were measured. The NO concentrations in the cell culture supernatants were determined by the Griess reagent kit (Beyotime Institute of Biotechnology, Haimen, China). The liquid supernatant (50 μL) was added to 100 μL Griess reagent, and then measured at 450 nm using a micro-plate reader (Spectramax 190, Molecular Devices, Sunnyvale, CA, USA). The TNF-*α* and IL-6 levels in cell culture supernatants were measured using enzyme-linked immunosorbent assay (ELISA) kits (ABclonal, Wuhan, China), according to the manufacturer’s instructions.

### 3.6. Potential Neuroprotective Activity of VOs in CORT-Induced PC12 Cells

Low-differentiated PC12 cells were cultured in a Roswell Park Memorial Institute (RPMI) 1640 medium (Pricella, Wuhan, China) supplemented with 10% FBS and 1% penicillin–streptomycin antibiotics (Pricella, Wuhan, China) at 37 °C in a 5% CO_2_-humidified environment. The cells were also treated with VOs at a concentration of 0–50 μg/mL for 24 h to evaluate their cytotoxicity with a CCK-8 assay.

To determine the potential neuroprotective activity of VOs, PC12 cells were seeded in 96-well plates and cultured for 24 h. Then, the cells were divided into the control group, CORT group, experiment group, and positive control group. The cells in the experiment and positive control groups were added to 400 μM CORT for 24 h, and then treated with 10 μg/mL VOs (VO-40, 50, and 60) or 5 μg/mL fluoxetine (FLU) for 24 h. The cells in the CORT group were first treated with 400 μM CORT for 24 h, and then replaced with an equal amount of the serum-free RPMI 1640 medium. The cells in control group did not undergo any treatment. After treatment for 48 h, the cell viability of all groups was measured using CCK-8 assay. And an LDH measurement of supernatant was performed using LDH cytotoxicity assay kit protocol (Elabscience, Wuhan, China), according to the manufacturer’s instructions.

### 3.7. Molecular Docking

Molecular docking analysis was employed to explore the interactions between selected compounds and proteins that can predict the binding patterns and affinities of compound–protein interactions. This study followed established protocols, as described in previous research [53,54]. To perform the molecular docking, the 3D structures of the selected potential active compounds, which have strong positive correlations with antifungal, anti-inflammatory, and potential neuroprotective properties, were obtained from the PubChem (https://pubchem.ncbi.nlm.nih.gov/, accessed on 23 December 2024). Five target proteins (chitin synthase, TNF-*α*, IL-6, GluN1, and GluN2B) were selected for molecular docking. Their structures were obtained from the PDB database (https://www.rcsb.org/, accessed on 23 December 2024) with PDB IDs 4WJW, 1EXT, 1ALU, 5H8Q, and 5EWM, respectively. These proteins were prepared by removing ligands and water present in them using PyMol v3.1 (DeLano Scientific, San Carlos, CA, USA). Then, the target proteins and the small molecules were hydrogenated before docking to ensure accuracy. Semi-flexible molecular docking was performed using Autodock v4.2.6 software (The Scripps Research Institute, La Jolla, CA, USA) to calculate the binding energies between the compounds and the target proteins. Before performing molecular docking on the novel compounds, the co-crystal inhibitors of each protein target were re-docked to validate the reliability of the docking protocol. Visual 2D and 3D representations of molecular interactions were generated using a Discovery Studio visualizer (Accelrys, San Diego, CA, USA) and PyMol v3.1, respectively.

### 3.8. Statistical Analysis

Statistical analyses were conducted using SPSS 23 (IBM, Armonk, NY, USA) with one-way analysis of variance, followed by Duncan’s multiple range test. Differences were considered statistically significant at a significance level of *p* < 0.05. All related graphs were produced using Origin 2019 (OriginLab, Northampton, MA, USA) and GraphPad Prism 10 (GraphPad Software, San Diego, CA, USA).

## 4. Conclusions

*C. japonica* solid wood boards are widely used in interior decoration and building construction. This study explored the VOCs in *C. japonica* solid wood boards to evaluate their impact on indoor air quality. A total of 58 VOCs were identified in the VOs from *C. japonica* solid wood boards with different tree ages, with sandaracopimarinol (**55**), ferruginol (**56**), abieta-7,13-diene (**51**), (+)-*δ*-cadinene (**23**), *cis*-cubenol (**36**), and sandaracopimarinal (**54**) as the major constituents. Twelve differential VOCs between VO-40, 50, and 60 were identified using PLS-DA and Spearman correlation analysis, such as *τ*-muurolol (**41**), sugiol (**58**), and 2,4-dimethylheptane (**5**). The VOs demonstrated antifungal activities against mold and wood-rotting fungi. Additionally, the VOs exhibited anti-inflammatory effects by inhibiting the production of NO, TNF-*α*, and IL-6 in LPS-induced RAW264.7 cells, as well as potential neuroprotective effects by increasing cell viability and reducing LDH release in CORT-induced PC12 cells. Correlation analysis revealed that compounds **54**, **55**, *β*-eudesmol (**39**), and **56** may be the main active compounds responsible for these effects. These findings highlight the potential of *C. japonica* solid wood boards to enhance indoor air quality and promote human health through the antifungal, anti-inflammatory, and neuroprotective properties of their volatile components. However, the current research on the anti-inflammatory and neuroprotective effects of the VOs is limited to the cellular level. To gain a deeper understanding of their mechanism of action and the long-term impacts on indoor environments and human health, further animal experiments and clinical studies are needed in the future. These efforts will provide a stronger foundation for the widespread application of *C. japonica* solid wood boards as high-quality, health-beneficial building materials.

## Figures and Tables

**Table 1:** Chemical composition of VOs in *C. japonica* solid wood boards with different tree ages.

No.	Compound	RT ^a^	RI ^b^	KI ^c^	Relative Content (%)		
					VO-40	VO-50	VO-60
	Alcohols				0.01	0.02	0.02
**1**	3-hexanol	4.72	798	797	0.01	0.01	0.01
**2**	2-hexanol	4.80	803	803	tr	0.01	0.01
	Aromatic hydrocarbons				0.31	0.39	0.29
**3**	(1*S*,4*S*)-4-isopropyl-1,6-dimethyl-1,2,3,4-tetrahydronaphthalen-1-ol	24.52	1659	1665	0.15	0.18	0.17
**4**	(1*R*,4*S*)-4-isopropyl-1,6-dimethyl-1,2,3,4-tetrahydronaphthalen-1-ol	24.87	1668	1675	0.16	0.20	0.12
	Hydrocarbons				0.01	0.01	tr
**5**	2,4-dimethylheptane	5.12	823	823	0.01	0.01	tr
**6**	3,7-dimethylundecane	13.74	1280	1222	–	0.01	–
	Ketones				0.02	0.01	0.02
**7**	3-hexanone	4.55	788	775	0.01	0.01	0.01
**8**	2-hexanone	4.64	793	791	0.01	0.01	0.01
	Oxygenated monoterpenes				0.02	–	0.01
**9**	linalool	9.82	1102	1103	–	–	0.01
**10**	*trans*-verbenol	10.80	1148	1148	0.01	–	–
**11**	verbenol	12.23	1214	1212	tr	–	tr
**12**	(−)-bornyl acetate	13.95	1289	1280	0.01	–	–
	Sesquiterpenes				12.78	10.46	9.99
**13**	*α*-cubebene	15.47	1354	1354	12.78	10.46	9.99
**14**	*α*-copaene	16.12	1381	1382	–	0.13	0.10
**15**	*cis*-*β*-copaene	16.45	1395	1433	0.16	0.05	0.03
**16**	*trans*-caryophyllene	17.18	1426	1435	0.13	0.16	0.11
**17**	*γ*-elemene	17.46	1438	1438	0.16	0.16	0.10
**18**	*cis*-muurola-4(15),5-diene	18.32	1474	1470	–	0.01	0.01
**19**	*γ*-muurolene	18.48	1481	1445	–	–	tr
**20**	*β*-selinene	18.76	1493	1493	0.31	0.19	0.24
**21**	*α*-muurolene	19.08	1504	1507	0.05	0.04	0.05
**22**	*β*-bisabolene	19.26	1510	1514	2.27	1.86	1.74
**23**	(+)-*δ*-cadinene	19.71	1525	1523	0.04	0.09	0.07
**24**	1,2,3,4,4a,7-hexahydro-1,6-dimethyl-4-(1-methylethyl)-naphthalene	19.96	1533	1530	9.27	7.53	7.15
**25**	(+)-*α*-cadinene	20.10	1538	1533	0.19	0.13	0.13
**26**	*α*-calacorene	20.28	1544	1542	0.05	–	0.04
**27**	germacrene B	20.73	1558	1567	0.08	0.08	0.11
**28**	*α*-corocalene	23.01	1624	1629	–	0.02	0.02
**29**	cadalene	25.18	1675	1688	–	0.01	–
	Oxygenated sesquiterpenes				23.89	24.33	22.36
**30**	(1*R*,4*R*,5*R*,6*R*,7*S*,10*R*)-4,10-dimethyl-7-propan-2-yltricyclo[4.4.0.01,5]decan-4-ol	18.95	1500	1494	4.93	4.52	3.72
**31**	cubebol	19.50	1518	1520	5.03	4.35	4.28
**32**	elemol	20.43	1548	1557	0.06	0.61	0.41
**33**	15-hydroxy-3-copaene	21.16	1572	1574	0.09	0.09	0.09
**34**	gleenol	21.61	1587	1595	1.06	0.79	0.76
**35**	humulene epoxide II	22.53	1612	1610	0.06	0.05	0.05
**36**	*cis*-cubenol	23.26	1630	1636	6.32	4.50	4.36
**37**	*γ*-eudesmol	23.41	1633	1637	–	0.74	0.61
**38**	epicubenol	23.84	1643	1653	4.87	3.27	3.46
**39**	*β*-eudesmol	24.16	1651	1656	0.30	2.09	1.70
**40**	*α*-eudesmol	24.31	1655	1656	–	2.01	–
**41**	*τ*-muurolol	24.32	1655	1660	–	–	1.98
**42**	*α*-cadinol	24.32	1655	1660	1.14	–	–
**43**	(7*S*)-7-isopropyl-4,10-dimethylene cyclodec-5-enol	25.91	1692	1695	–	0.09	0.06
**44**	((4a*S*,8*S*,8a*R*)-8-isopropyl-5-methyl-3,4,4a,7,8,8a-hexahydronaphthalen-2-yl)methanol	26.21	1699	1782	0.03	0.04	0.02
**45**	cyperotundone	27.29	1722	1717	–	0.03	–
**46**	cryptomerione	27.47	1726	1734	–	1.00	0.77
**47**	proximadiol	31.48	1814	1822	–	0.16	0.09
	Diterpenes				17.94	10.70	12.40
**48**	sandaracopimaradiene	35.08	1965	1973	1.40	0.91	0.88
**49**	palustradiene	35.78	2016	2036	0.69	–	–
**50**	7-isopropyl-1,1,4a-trimethyl-1,2,3,4,4a,9, 10,10a-octahydrophenanthrene	36.27	2062	2069	1.41	1.26	1.24
**51**	abieta-7,13-diene	36.57	2090	2082	13.66	8.20	9.84
**52**	neoabietadiene	37.25	2157	2171	0.78	0.32	0.44
	Oxygenated diterpenes				30.79	35.72	35.11
**53**	(+)-manoyl oxide	35.49	1992	2007	0.04	0.06	0.05
**54**	sandaracopimarinal	37.66	2199	2213	3.23	6.04	5.93
**55**	sandaracopimarinol	38.41	2266	2288	13.74	17.14	17.59
**56**	ferruginol	39.35	2342	2336	11.29	10.64	10.23
**57**	abietyl alcohol	40.36	2414	2389	0.45	0.57	0.39
**58**	sugiol	41.34	2474	2660	2.04	1.27	0.93
	Identification				85.78	81.64	80.21
	Yields of VOs (%)				1.14	1.17	1.29

^a^ RT, retention time; ^b^ RI calculated from retention times in relation to those of a series of C_7_–C_40_ *n*-alkanes on a HP-5MS column; ^c^ Kovats index (KI) corresponds to data reported previously in the National Institute of Standards and Technology (NIST) database or in the literature on a HP-5MS column; tr: trace (<0.005%); –: compounds not detected.

**Table 2:** Potential differential VOCs in VOs from *C. japonica* solid wood boards with different tree ages identified by two statistical methods.

Compound	VIP Value (PLS-DA)	Spearman Correlation Coefficient
*τ*-muurolol (**41**)	1.51	0.85
cadalene (**29**)	1.40	–
sugiol (**58**)	1.31	0.95
*trans*-caryophyllene (**16**)	1.25	–
*α*-eudesmol (**40**)	1.22	–
abietyl alcohol (**57**)	1.19	–
*cis*-cubenol (**36**)	1.17	0.63
sandaracopimaradiene (**48**)	1.17	0.84
DPYD (**30**)	1.16	–
gleenol (**34**)	1.11	–
*trans*-verbenol (**10**)	1.10	0.85
*α*-cadinol (**42**)	1.10	0.85
palustradiene (**49**)	1.10	0.85
*α*-muurolene (**21**)	1.09	0.63
2,4-dimethylheptane (**5**)	1.09	0.95
(+)-*δ*-cadinene (**23**)	1.09	0.58
epicubenol (**38**)	1.06	0.69
neoabietadiene (**52**)	1.05	–
abieta-7,13-diene (**51**)	1.04	0.79
germacrene B (**27**)	1.03	–
*α*-copaene (**14**)	–	0.79
*γ*-muurolene (**19**)	–	0.74
(−)-bornyl acetate (**12**)	–	0.65
*cis*-muurola-4(15),5-diene (**18**)	–	0.65
linalool (**9**)	–	0.65
HDMN (**24**)	–	0.63
*β*-bisabolene (**22**)	–	0.63
15-hydroxy-3-copaene (**33**)	–	0.58

–: not detected; DPYD: 1*R*,4*R*,5*R*,6*R*,7*S*,10*R*)-4,10-dimethyl-7-propan-2-yltricyclo[4.4.0.01,5]decan-4-ol; HDMN: 1,2,3,4,4a,7-hexahydro-1,6-dimethyl-4-(1-methylethyl)-naphthalene.

**Table 3:** Antifungal and antibacterial activities of VO-40, 50, and 60 in *C. japonica* solid wood boards.

Sample	MIC (mg/mL) ^a^						
	*A. fumigatus*	*A. niger*	*G. trabeum*	*P. fomentarius*	*S. aureus*	*E. coli*	*P. aeruginosa*
VO-40	1.250	5.00	2.50	2.50	>10	>10	>10
VO-50	0.312	2.50	2.50	2.50	>10	>10	>10
VO-60	0.625	5.00	1.25	5.00	>10	>10	>10
Ketoconazole ^b^	62.5 ^c^	3.9 ^c^	0.49 ^c^	0.98 ^c^	–	–	–
Tetracycline ^b^	–	–	–	–	15.6 ^c^	3.9 ^c^	62.5 ^c^

^a^ MIC, minimum inhibitory concentration of VOs at which fungi and bacteria were ungrown; ^b^ positive control; ^c^ in μg/mL; –: not detected.

**Table 4:** Docking results of active compounds in VOs with chitin synthase, TNF-*α*, IL-6, GluN 1, and GluN 2B.

No.	Compound	4WJW	1EXT	1ALU	5H8Q	5EWM
		Free Binding Energy (kcal/mol)				
**39**	*β*-eudesmol	–5.24	–5.62	–5.43	–5.58	–5.00
**54**	sandaracopimarinal	–5.64	–5.31	–6.69	–5.95	–6.04
**55**	sandaracopimarinol	–6.72	–6.41	–5.41	–4.99	–5.71
**56**	ferruginol	–6.35	–5.76	–5.96	–6.14	–5.43

## Data Availability

The data are contained within the article.

## Abbreviations

The following abbreviations are used in this manuscript:

VOCs	Volatile organic compounds
VOs	Volatile oils
SFE-CO_2_	Supercritical CO_2_ fluid extraction
GC-MS	Gas chromatography–mass spectrometry
RIs	Retention indices
NIST	National Institute of Standards and Technology
KIs	Kovats indices
PLS-DA	Partial least squares-discriminant analysis
MIC	Minimum inhibitory concentration
DMSO	Dimethyl sulfoxide
LPS	Lipopolysaccharide
DMEM	Dulbecco’s modified Eagle’s medium
FBS	Fetal bovine serum
CCK-8	Cell Counting Kit-8
DEX	Dexamethasone
NO	Nitric oxide
TNF-*α*	Tumor necrosis factor-*α*
IL-6	Interleukin 6
ELISA	Enzyme-linked immunosorbent assay
CORT	Corticosterone
RPMI	Roswell Park Memorial Institute
FLU	Fluoxetine
LDH	Lactate dehydrogenase
VIP	Variable importance in projection
A*β*	Beta-amyloid
DPYD	(1*R*,4*R*,5*R*,6*R*,7*S*,10*R*)-4,10-di-methyl-7-propan-2-yltricyclo[4.4.0.01,5]decan-4-ol
HDMN	1,2,3,4,4a,7-hexahydro-1,6-di-methyl-4-(1-methylethyl)-naphthalene
ITO	7-isopropyl-1,1,4a-trimethyl-1,2,3,4,4a,9,10,10a-octahydrophenanthrene
